# Vascular endothelial growth factor-B expression in postischemic rat brain

**DOI:** 10.1186/2045-824X-5-8

**Published:** 2013-04-20

**Authors:** Lin Xie, Xiaoou Mao, Kunlin Jin, David A Greenberg

**Affiliations:** 1 Buck Institute for Research on Aging, 8001 Redwood Boulevard, Novato, CA 94945, USA; 2 Department of Pharmacology & Neuroscience, University of North Texas, 3500 Camp Bowie Boulevard, Fort Worth, TX 76107, USA

**Keywords:** Vascular endothelial growth factor-B (VEGF-B), Stroke, Ischemia

## Abstract

**Background:**

Vascular endothelial growth factor-B (VEGF-B) protects against experimental stroke, but the effect of stroke on VEGF-B expression is uncertain.

**Methods:**

We examined VEGF-B expression by immunohistochemistry in the ischemic border zone 1–7 days after middle cerebral artery occlusion in rats.

**Results:**

VEGF-B immunoreactivity in the border zone was increased after middle cerebral artery occlusion and was associated with neurons and macrophages/microglia, but not astrocytes or endothelial cells.

**Conclusions:**

These findings provide additional evidence for a role of VEGF-B in the endogenous response to cerebral ischemia.

## Background

Vascular endothelial growth factor (VEGF-A), identified originally as an angiogenic [1] and vascular permeability [2] factor, also exhibits direct neurotrophic [3] and neuroprotective [4] effects. VEGF-A reduces infarct size in acute experimental stroke [5] and has been implicated in postischemic brain repair [6]. Several studies have documented the induction of VEGF-A RNA or protein expression in ischemic brain [7-15], suggesting that VEGF-A may participate in the brain’s endogenous response to ischemic injury. Induction of VEGF-A is observed hours to days after the onset of focal brain ischemia, is prominently associated with the ischemic border zone, and occurs in a variety of cell types, including neurons, astrocytes, endothelial cells, and macrophages/microglia. Upregulation of the two principal VEGF-A tyrosine kinase receptors, VEGFR-1 and VEGFR-2, occurs in concert with VEGF-A induction [7,9,11-14].

In addition to VEGF-A, several other VEGF family members have been described, including VEGF-B, VEGF-C, and placenta growth factor (PlGF). Of these, a protective effect in stroke has been shown most clearly for VEGF-B. In homozygous VEGF-B-knockout mice, permanent occlusion of the middle cerebral artery (MCA) produced larger infarcts and more severe neurological deficits than in heterozygous knockout or wild-type mice, suggesting that VEGF-B is normally protective against ischemia [16]. Moreover, intracerebral administration of VEGF-B reduced ischemic cell death after MCA occlusion in both VEGF-B-knockout and wild-type mice [17].

Induction of both VEGF-C [13,18] and PlGF [19] by cerebral ischemia has been reported, but the effect on VEGF-B is controversial. One study found increased VEGF-B immunoreactivity in the ischemic border zone 24 hr after permanent MCA occlusion in mice [17], whereas another reported reduced abundance of VEGF-B protein 24 hr after transient MCA occlusion in rats [20]. To address this inconsistency, we measured VEGF-B protein expression and its distribution for up to 1 wk after MCAO in rats.

## Methods

Animal experiments were approved by local committee review and conducted according to the National Institutes of Health guidelines. Male Sprague–Dawley rats (280–310 g) were anesthetized with 4% isoflurane in 70% N_2_O and 30% O_2_ using a mask. A midline incision was made in the neck, the right external carotid artery was carefully exposed and dissected, and a 3–0 monofilament nylon suture was inserted from the external carotid artery into the right internal carotid artery to occlude the origin of right middle cerebral artery (MCA). After 90 minutes of occlusion, the suture was removed to allow reperfusion, the external carotid artery was ligated, the wound was closed, and the rats were allowed to awaken. Rectal temperature was maintained (37.0 ± 0.5°C) with a heating pad and lamp and blood pressure was monitored.

Rats were re-anesthetized 1, 3 or 7 days after MCA occlusion and perfused through the heart with 4% paraformaldehyde in phosphate-buffered saline (PBS, pH 7.4). Brains were dehydrated in graded ethanol, cleared in xylene, and paraffin-embedded. Seven micrometer-thick serial coronal sections were cut and mounted on glass slides, which were dried overnight at 42°C. Sections were then washed in PBS for 20 min, permeabilized in 0.2% Triton in Tris-buffered saline for 1 h, blocked using 5% normal goat serum with 0.2% Triton in Tris-buffered saline for 1 h, and incubated with primary antibody in blocking buffer at 4°C overnight. The primary antibodies used were: rat anti-VEGF-B (1:50, Santa Cruz), rabbit anti-MAP2 (1:500, Chemicon), rabbit anti-CD11b (1:200, Abcam), rabbit anti-GFAP (1:1,000, Sigma), and rabbit anti-von Willebrand factor (vwF) (1:500, Sigma).

For diaminobenzidine (DAB) staining, sections stained for VEGF-B as above were washed in PBS containing 0.1% Tween 20 for 1 h, incubated with biotinylated anti-mouse IgG secondary antibody (1:200, Vectastain Elite ABC, Vector) in blocking buffer for 1 h, and placed in avidin–peroxidase conjugate (Vector) for 1 h. The horseradish peroxidase reaction was detected with 0.05% DAB and 0.03% H_2_O_2_. Processing was stopped with H_2_O and sections were imaged using a Nikon E300 epifluorescence microscope.

For double-label immunofluorescence staining, sections stained with the primary antibodies listed above were washed in PBS containing 0.1% Tween 20 for 1 h, incubated with secondary antibody in blocking buffer for 1 h, washed in PBS for 1 h, and mounted using Prolong Gold with DAPI (4',6-diamidino-2-phenylindole, Invitrogen). Secondary antibodies were Alexa Fluor-conjugated donkey anti-mouse or anti-rabbit IgG (1:200, Molecular Probes). Sections were imaged on a laser scanning confocal microscope (LSM 510, Carl Zeiss).

## Results

**Figure 1:**
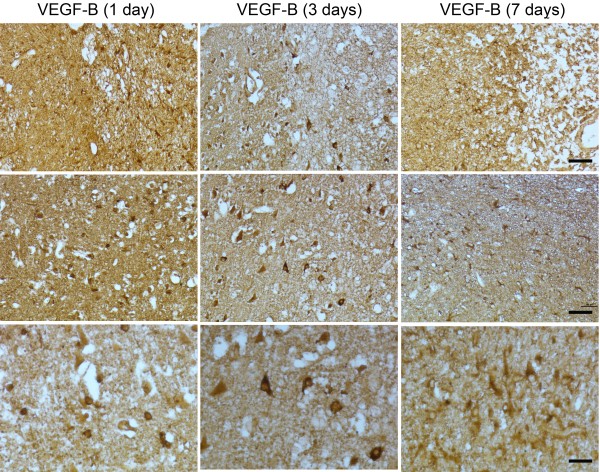
**VEGF-B immunoreactivity in cerebral cortex 1–7 days after MCAO.** Top row, top right corner is in the direction of the ischemic core (bar, 50 μm). Middle row, ischemic border zone (bar, 50 μm). Bottom row, ischemic border zone (bar, 20 μm).

**Figure 2:**
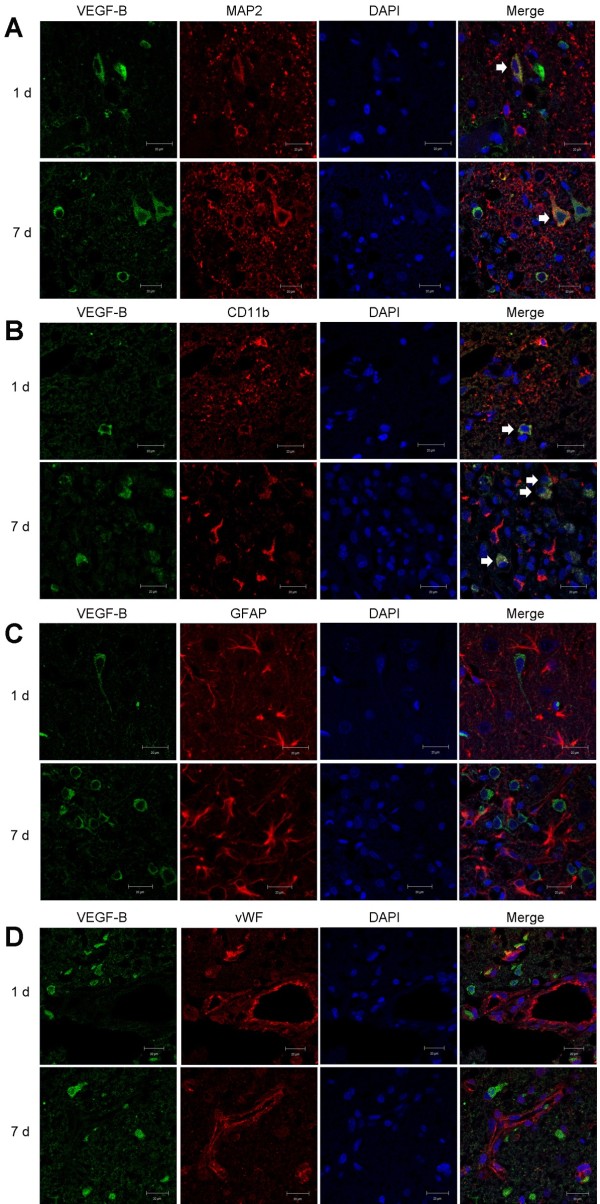
**VEGF-B (left column, green), cell-type marker (second column from left, red), DAPI (third column from left, blue), and merged (right column, white arrows) staining of cells in the cerebral cortex ischemic border zone 1 and 7 days after MCAO.** Cell-type markers are (**A**) MAP2 (neurons), (**B**) CD11b (macrophages/microglia), (**C**) GFAP (astrocytes), and (**D**) vWF (endothelial cells). Colocalization of VEGF-B with MAP2 and CD11b, but not GFAP or vWF, was detected. Bars, 20 μm.

DAB-stained sections through the ischemic hemisphere of rat brains showed increased VEGF-B immunoreactivity, primarily in the ischemic border zone, at 1, 3, and 7 days after MCAO (Figure 1). This immunoreactivity was associated with cell bodies of round and polygonal cells and was more pronounced at 1 and 3 than at 7 days. Double-label immunohistochemistry was done on 1- and 7-day postischemic brains to identify cell types associated with VEGF-B expression. These sections showed colocalization of VEGF-B with the neuronal marker MAP2 and the macrophage/microglial marker CD11b, but not the astrocytic marker GFAP nor the endothelial cell marker vWF (Figure 2). VEGF-B was detected in ~50% of MAP2-immunopositive cells in the border zone.

## Discussion

The main finding of this study is that VEGF-B protein is upregulated in neurons and inflammatory (macrophage/microglial) cells of the ischemic border zone from 1–7 days after MCAO in rats. This is in accord with previous findings pertaining to other VEGF family members (VEGF-A and VEGF-C) and VEGF receptors (VEGFR-1, VEGRR-2 and VEGFR-3) [7-15,18,19]. It is also in agreement with one prior report of increased VEGF-B expression after cerebral ischemia [17], but contrasts with another study indicating that VEGF-B expression declines in this setting [20]. Methodologic differences that might account for the discrepancy between this latter study and ours include the use of different rat strains (Wistar vs. Sprague Dawley), 180- vs. 90-min MCAO, single (24 h) vs. multiple (1, 3 and 7 days) postischemic time points, and analysis of the whole hemisphere by western blot vs. the ischemic border zone by immunohistochemistry. The latter is especially likely to have been a factor because whole-hemisphere sections include the ischemic core, where protein synthesis is impaired, and cannot resolve regional heterogeneity of protein expression [21].

Our finding of increased VEGF-B expression in ischemic brain is consistent with other examples of VEGF-B induction by neuropathological processes. These include cortical cold injury in rats [22], motor neuron degeneration in SOD1^G93A^ transgenic mice [23], a cell-culture model of Parkinson’s disease [24], and type IIB focal cortical dysplasia in humans [25]. VEGF-B deletion exacerbates motor neuron degeneration [23] and paclitexel-induced sensory neuronopathy [26] in mice, which resembles our earlier finding that VEGF-B deletion worsens outcome from experimental stroke in mice [16], and provides additional support for a neuroprotective role.

Two questions raised by this study are what couples cerebral ischemia and VEGF-B induction and how VEGF-B induction affects the ischemic brain. Regarding the stimulus to induction, VEGF-B differs from VEGF-A in that the former does not contain a hypoxia-response element in the promoter region [27] and is not transcriptionally induced by hypoxia [28]. Consequently, some other mechanism must be involved in its induction by ischemia.

How induction of VEGF-B affects outcome from stroke is unclear, but VEGF-B has been shown to be an antiapoptotic [17] and cell-survival [29] factor in several tissues. Unlike VEGF-A, it is not thought to play a prominent role in angiogenesis [30], and operates primarily via interaction with VEGFR-1, rather than the angiogenesis-associated VEGFR-2 targeted primarily by VEGF-A [31]. In the retina, VEGF-B signaling through VEGFR-1 inhibits proapoptotic gene expression, as well as retinal ganglion cell death from oxidative injury, axotomy, and excitotoxicity [17]. Similar mechanisms might therefore mediate the effects of VEGF-B in stroke. In addition, VEGF-B stimulates adult neurogenesis [32], which may promote a more favorable outcome after stroke [33,34].

## Conclusions

VEGF-B, like other VEGF family members, is induced by experimental stroke, and may therefore contribute to endogenous adaptive mechanisms that limit ischemic brain injury.

## Abbreviations

DAB: Diaminobenzidine; DAPI: 4',6-diamidino-2-phenylindole; GFAP: Glial fibrillary acidic protein; MAP2: Microtubule-associated protein 2; MCA: Middle cerebral artery; PlGF: Placenta growth factor; VEGF: Vascular endothelial growth factor; VEGFR: Vascular endothelial growth factor receptor; vwF: Von Willebrand factor

## Competing interests

The authors declare that they have no competing interests.

## Authors’ contributions

LX performed animal surgery and immunohistochemistry and helped write the paper. XM performed immunohistochemistry. KJ helped design the research and analyze the data. DAG designed the research, analyzed the data, and wrote the paper. All authors read and approved the final version of the manuscript.
